# Safety of EndoAnchors in real-world use: A report from the Manufacturer and User Facility Device Experience database

**DOI:** 10.1177/1708538119844041

**Published:** 2019-04-16

**Authors:** Reza Masoomi, Emily Lancaster, Alexander Robinson, Ethan Hacker, Zvonimir Krajcer, Kamal Gupta

**Affiliations:** 1 Division of Cardiovascular Medicine, The University of Kansas Medical Center, Kansas City, KS, USA; 2 Division of Internal Medicine, Cornell University, Ithaca, NY, USA; 3 Division of Cardiovascular Medicine, Texas Heart Institute, Houston, TX, USA

**Keywords:** Endovascular aneurysm repair, Manufacturer and User Facility Device Experience, endoleak, EndoAnchor

## Abstract

**Objectives:**

A hostile proximal neck anatomy is the most common cause of abdominal aorta endovascular aneurysm repair failure leading to a higher risk of device migration, proximal type I endoleak, and subsequent open surgical repair. Endostapling is a technique to attain better fixation of the endograft to the aortic wall, and the only available device in the USA is Aptus Heli-FX EndoAnchor system (Medtronic Vascular, Santa Rosa, CA, USA). Preliminary data have shown efficacy and safety of its use, and the aim of this study is to assess device-related adverse events in real-world clinical use.

**Methods:**

We quarried data from the publicly available Manufacturer and User Facility Device Experience database to identify Aptus Heli-FX EndoAnchor system-related adverse reports in endovascular aneurysm repair since FDA approval till August 31, 2017. An estimate of total devices implanted in the United States was quoted around 7,000 (Medtronic marketing internal data).

**Results:**

Our query identified 229 separate reports, of which there were 85 adverse events (1.2% of the estimated EndoAnchor systems used). The most common adverse events were device dislodgement/fracture (65) and applicator malfunction (20).

**Conclusion:**

In early post-FDA approval use in a real-world setting, the EndoAnchor system is associated with a low rate of adverse events. Device dislodgement and embolization remain the most common adverse events. With increasing use of these devices in more difficult anatomy, careful patient selection and careful attention to technique may help to reduce these events even further.

## Introduction

Endovascular aneurysm repair (EVAR) is the preferred treatment modality for an abdominal aortic aneurysm (AAA). A significant barrier for EVAR is the presence of hostile proximal aortic neck anatomy.^1–3^ Several anatomic features are considered unfavorable for EVAR, which lead to a higher risk of device migration, proximal type I endoleak, and subsequent or rescue open surgical AAA repair.^4^ Therefore, these group of patients face poor outcomes after endovascular repair due to endoleak or graft migration.

These challenges have led to developing new techniques/devices to prevent and treat device migration and proximal type I endoleak in patients with hostile neck anatomy. One such technique is endostapling or endotacking, where screw-like anchors are used to attain better approximation and fixation of the endograft to the aortic wall at the proximal neck.^2,5^ Currently, the only device approved for clinical use in the USA is the Aptus Heli-FX EndoAnchor system (Medtronic Vascular, Santa Rosa, CA, USA). This device was approved for use by the FDA in November 2011. Since its approval, there has been a significant increase in EndoAnchor use in the USA with recent publications reporting that EndoAnchors do decrease the incidence of endoleak and adverse outcomes in those with complex neck anatomy.^6–10^

Although the pivotal study for the Aptus Heli-FX EndoAnchor system showed an extremely low risk of complications, real-world data on device use and safety is lacking. The main objective of this study was to assess the safety of EndoAnchor use in the routine clinical practice. We consulted the Manufacturer and User Facility Device Experience (MAUDE) database to assess device-related adverse events since FDA approval in the US.^11^

## Methods

The MAUDE database is a searchable online database of medical device reports received by the FDA. Most reports originate from manufacturers (mandatory) and approximately 5% are submitted by user facilities (voluntary) including hospitals and clinics. The FDA requires manufacturers to report adverse events that are communicated to them verbally or in writing. Manufacturers have been reporting events since 1996 and this database is updated on a monthly basis. These reports serve as a passive surveillance tool to monitor device performance and adverse events associated with device use.

For the purpose of this study, we queried the MAUDE database for all events involving the name “Aptus Heli-FX EndoAnchor system” since FDA approval date (November 2011) till August 31, 2017. Accurate data on the number of devices implanted in the USA is not available publically. We obtained an estimate of device systems used through direct correspondence with the manufacturer. This figure was quoted as around 7000 systems used (Medtronic data on file). However, it still approximates the volume of device use in the USA.

MAUDE data does not include any identifying patient information and therefore the study was exempted from our institution’s human subjects committee review. Two members of our research team reviewed all reports independently and reports were categorized as residual endoleak, dislodgement or fracture of the EndoAnchors, air embolism, guide/applier malfunction, and other adverse events. Reports deemed unrelated to the device were not included. The characterization of each report was compared between the two investigators and any discrepancy was resolved by consensus. There were a few reports of deaths associated with EndoAnchor use. However, a review of each case indicated that most were not related to device use and these were not included as device-related complications. There were also a few reports of access site vascular injury during the EVAR procedure. After reviewing these reports, we concluded that these were likely related to the EVAR procedure itself and not related to EndoAnchor use and thus were not included as device-related adverse events.

Adverse events were reported by counts (%) and descriptive statistics were used to report the clinical adverse events. More than one complication categorization could be assigned to each report. All statistical analyses were conducted using SPSS 22.0 (IBM, Chicago, IL).

## Results

**Figure 1.**
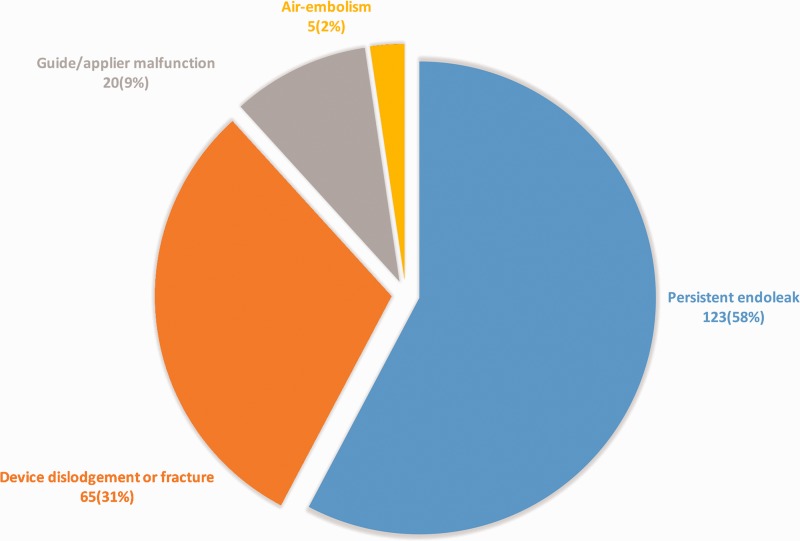
Reported events by type.

Since FDA clearance of the device, our query of the MAUDE database produced 296 reports. Of these, 16 reports were duplicate, 47 reports were not relevant to the use of the device (such as hospitalization for uncontrolled hypertension, failure to thrive, hospital acquired infection, etc.), and 4 reports involving thoracic aortic aneurysm repair and thus all these were excluded. Thus, we identified 229 separate reports describing possible adverse events (Figure 1). The mean time between event occurrence and date reported to the FDA was 238 days.

**Figure 2.**
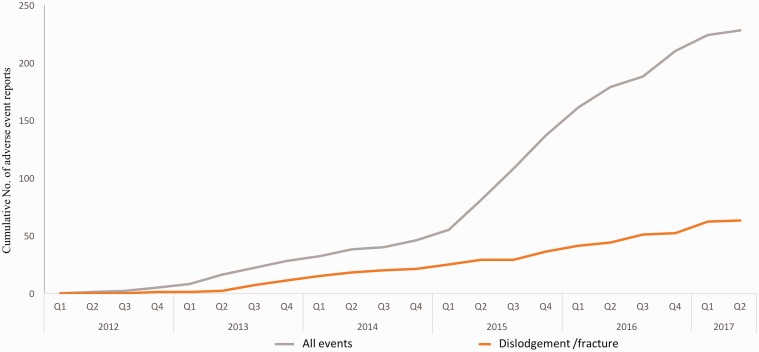
Cumulative adverse events related to EndoAnchor implantation.

The most commonly reported event was failure to resolve or recurrence of a type IA endoleak (123 reported cases constituting 58% of reports and 1.7% of estimated total device systems used). The next most commonly reported event was device dislodgement or fracture (65 reports constituting 31% of reports and 0.9% of estimated total device systems used). The fractured device embolized to renal artery in two cases, hypogastric artery in one case, and the flow divider of the endograft in one case. Other less common adverse events were five reports of air embolism, and 20 reports of guide/applier malfunction (often requiring removal and use of a new applier). No adverse long-term clinical consequences were reported from these cases of air embolism or applicator malfunctions. Figure 2 shows the cumulative adverse events reported since early 2015. This has occurred with an increased EndoAnchor usage per direct communication from manufacturer (penetration of EndoAnchors in total EVAR + TEVAR cases in the US increased from 2.1% to 4.2% in US between 2014–2017) and thus does not necessarily represent an increase in the adverse event rate. Since there is a delay between occurrence date and reception date of the primary report, and we do not have the exact number of devices being used per year, an annual complication incidence rate cannot be accurately ascertained.

There were total 27 deaths reported during index procedure hospitalization or on follow-up. In a database such as MAUDE, it is not possible to ascertain causality of these deaths. We reviewed the details of each death report from the information available. Of those, there were 15 reports with unknown etiology or unrelated to the index procedure per our adjudication, and 12 reports were thought to be related to the index EVAR procedure rather than EndoAnchor use. However, there was one report where multiple tiny holes were seen at the site of EndoAnchor insertion in fabric at the time of explant (and these were thought by the reporting physician to have contributed to worsening endoleak).

## Discussion

The main finding of this study is that the reported adverse event rate, in the early real-world (post-FDA approval) usage of the EndoAnchors, is low and only slightly above the reported rate in the clinical trials.

This is the first study to report on adverse events related to EndoAnchor use in the real-world setting. The Aneurysm Treatment Using the Heli-FX Aortic Securement System Global Registry (ANCHOR) clinical trial started recruiting from April 2012,^12^ and primary results of this registry including 319 patients showed that implantation of EndoAnchor was technically successful in 98.1% patients with no ruptures, migrations, or open surgical conversions, and the frequency of fracture was only 0.3%. Since clinical trials have expert physician users and carefully screened patients, the concern is always that such results may not be replicated in the real-world setting due to a diverse user expertise, more complex patients and adverse patient anatomy. Since its approval for clinical use in the USA, it is estimated that about 7000 EndoAnchor systems have been used (direct communication from manufacturer). This represents a doubling of use in the past three years (2.1% of cases in 2014 to 4.2% of cases in 2017).

The most common reported event type in our study was persistence or recurrence of type I endoleak. Although it can represent an unsuccessful procedure, it cannot be considered a true adverse event. Therefore, the true reported adverse event types were those of EndoAnchor fracture/dislodgment and applicator malfunction in order of decreasing frequency with a total number of 85 reports (1.2% of the estimated EndoAnchor systems used). It is slightly higher than ANCHOR study, albeit no significant eventual clinical harm was reported in majority of cases and the number of cases where EndoAnchors could not be retrieved or caused vascular injury in case of fracture/dislodgement was very low. There were five cases of air embolism reported with use of EndoAnchors. Although air embolism is rare and it can also happen with any stent graft deployment as likely due to inadequate flushing, and it is not necessarily related to the use of EndoAnchors.^13,14^ This complication can be prevented by meticulous care to catheter flushing, care during device insertion, and over-flushing particularly thoracic devices with possible supplementary use of CO_2_.

Encountering more events in real-world population is likely a result of more patient complexity, less user expertise, emergent or urgent EVAR procedures, and higher use in non-elective procedures. Further, EndoAnchors are being used in patients with ever increasing adverse neck anatomy. Therefore, the relatively low complication rate in real-world setting is reassuring.

A prerequisite to use of EndoAnchors for operators is to familiarize themselves with the indications and technical details for the device (Table 1).^12^ They should also undergo the training and professional guidance until they gain expertise in using EndoAnchors. The device manufacturer provides training and in procedure support which are critical in helping identify the appropriate patients and those with lower chances of success and higher complication risk.

**Table 1.** Indications and contraindications for EndoAnchor use.

Contraindications to use
Mural thrombus >2 mm thick and 180° of circumference or severe circumferential calcification
Endologix Powerlink endograft
Loss of graft apposition
Indications to use
Normal neck anatomy with higher risk of re-intervention (comorbidities, potential loss of follow-up or young patients)
Hostile neck anatomy (concerns for implant stability, challenging neck or difficult landing)
Revision (acute type I endoleaks, late type I endoleaks or migration)

There have been discussions and suggestions to increase the prophylactic use of EndoAnchors in the presence of difficult neck anatomy and our study provides some level of reassurance on the safety of these devices.^10^ Further, a detailed study of cases where the device failed to close the type I endoleak should be done to see if technique modification and better case selection may help.

## Limitations

The MAUDE registry unfortunately does not provide the information on aortic neck anatomy and patients’ characteristics. It is possible that the great majority of complications occurred when devices used in non-indicated or situations with relative contraindications. We could not calculate true incidence of complications since we do not have exact data regarding rate of device use. The information submitted to MAUDE database has several limitations including possibility of inaccurate or incomplete data, and most importantly under-reporting. Also, there is a time delay between the event date and report date to FDA (days to months), which can lead to underestimation of overall incidence of adverse events. For these reasons, the incidence of adverse events cannot be accurately determined through the MAUDE database. However, the MAUDE data does provide an objective assessment of the real-world complications and adverse patient events.

## Conclusion

In early post-FDA approval use in clinical practice, the EndoAnchor system is associated with a low rate of adverse events. Device dislodgement and embolization remain the most common adverse events followed by applicator malfunction. Further, most adverse events occurred in non-elective cases. With increasing use of these devices in more difficult anatomy, careful patient selection and careful attention to technique may help to reduce these adverse events even further.
